# Experimental Design and Data Analysis Issues Contribute to Inconsistent Results of C-Bouton Changes in Amyotrophic Lateral Sclerosis

**DOI:** 10.1523/ENEURO.0281-16.2016

**Published:** 2017-01-18

**Authors:** S. Shekar Dukkipati, Aouatef Chihi, Yiwen Wang, Sherif M. Elbasiouny

**Affiliations:** 1 Department of Neuroscience, Cell Biology, and Physiology, Boonshoft School of Medicine and College of Science and Mathematics, Wright State University, Dayton, Ohio 45435; 2 Department of Biomedical, Industrial, and Human Factors Engineering, College of Engineering and Computer Science, Wright State University, Dayton, Ohio 45435

**Keywords:** ALS, amyotrophic lateral sclerosis, C-bouton, cholinergic synapse, motoneuron, *SOD1-G93A*

## Abstract

The possible presence of pathological changes in cholinergic synaptic inputs [cholinergic boutons (C-boutons)] is a contentious topic within the ALS field. Conflicting data reported on this issue makes it difficult to assess the roles of these synaptic inputs in ALS. Our objective was to determine whether the reported changes are truly statistically and biologically significant and why replication is problematic. This is an urgent question, as C-boutons are an important regulator of spinal motoneuron excitability, and pathological changes in motoneuron excitability are present throughout disease progression. Using male mice of the *SOD1-G93A* high-expresser transgenic (*G93A*) mouse model of ALS, we examined C-boutons on spinal motoneurons. We performed histological analysis at high statistical power, which showed no difference in C-bouton size in *G93A* versus wild-type motoneurons throughout disease progression. In an attempt to examine the underlying reasons for our failure to replicate reported changes, we performed further histological analyses using several variations on experimental design and data analysis that were reported in the ALS literature. This analysis showed that factors related to experimental design, such as grouping unit, sampling strategy, and blinding status, potentially contribute to the discrepancy in published data on C-bouton size changes. Next, we systematically analyzed the impact of study design variability and potential bias on reported results from experimental and preclinical studies of ALS. Strikingly, we found that practices such as blinding and power analysis are not systematically reported in the ALS field. Protocols to standardize experimental design and minimize bias are thus critical to advancing the ALS field.

## Visual Abstract

**Figure:**
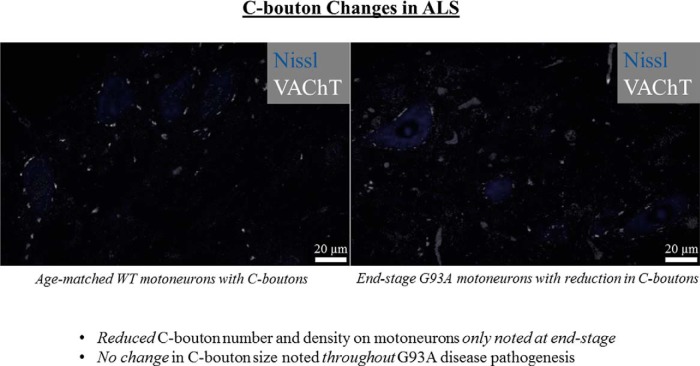

## Significance Statement

Cholinergic boutons (C-boutons) are cholinergic synaptic inputs that have been implicated as an important regulator of motoneuron excitability. Pathological changes in the size of C-boutons have been previously characterized in disease models of ALS, with conflicting results reported. This issue is critical, as the dysregulation of motoneuron excitability is present throughout disease progression. We show here that reported changes in C-bouton size, number, and density—thought to be either disease-related mechanisms or compensatory processes to maintain cell excitability—cannot be replicated. Furthermore, we provide evidence suggesting the possible reasons for the failure of replication due to variability in experimental design and analysis practices. Additionally, we show that such variability is widespread in other animal studies of ALS and propose practices for improved consistency.

## Introduction

Amyotrophic lateral sclerosis (ALS) is a progressive neurodegenerative disease that is characterized by selective degeneration and death of upper and lower motoneurons in the cortex, brainstem, and spinal cord, leading to muscle weakness, atrophy, paralysis, and death (Charcot, 1874; Rowland and Shneider, 2001). After decades of research, there are reports of many disease changes and many hypotheses on pathogenesis; yet, the field lacks the clear mechanistic understanding needed to prevent or impede motoneuron degeneration in ALS. The only Food and Drug Administration-approved treatment for ALS patients is riluzole, which extends survival marginally (∼3 months; Bensimon et al., 1994).

Several mutations have been identified in ALS patient populations and recreated within transgenic animal models. Among those is the G93A superoxide dismutase 1 (*G93A*) model (with >25 copies; Gurney et al., 1994), which is the most widely characterized and extensively studied model of ALS (Turner and Talbot, 2008). *G93A* mice develop a phenotype similar to that of ALS patients, including motor impairment, axonal loss, motoneuron death, muscle atrophy, and limb weakness (Fischer et al., 2004); and riluzole was developed through studies of the *G93A* line. We used this disease model, with wild-type (WT) transgenic controls, in the present study.

Cholinergic bouton (C-bouton) size changes represent an important, but disputed, topic within the field. Recently, a large number of studies have examined potential changes in C-bouton size during ALS disease progression in male mice of the *G93A* line (summarized in Table 1). However, the reported results have been conflicting: various studies report enlargement (Pullen and Athanasiou, 2009; Herron and Miles, 2012; Saxena et al., 2013), no change (Pullen and Athanasiou, 2009; Herron and Miles, 2012; Milan et al., 2015), or shrinkage (Milan et al., 2015) of C-boutons at various disease stages. Because C-bouton changes could potentially contribute to excitability dysregulation of motoneurons in ALS, reconciliation of these findings is a critical issue. If these changes are verified, they would improve our understanding of the mechanisms underlying disease pathogenesis and could represent an important therapeutic target for restoring normal excitability to degenerating motoneurons. Thus, our objectives were (1) to determine whether variations in study design or analysis practices could underlie the conflicting results reported in literature and (2) to resolve this research question by examining whether we can replicate any of the changes reported in male mice of the *G93A* line on C-bouton size. Our hypotheses were (1) that variations in study design and data analysis would impact our study results and (2) that a large-sample study with high statistical power would demonstrate C-bouton size changes in a mouse model of ALS, compared with WT mice. Both objectives are important because conflicting reports of study results impede understanding of this and other ALS pathophysiologies, thus delaying the development of effective treatments.

**Table 1.** Recent literature examining C-bouton size in ALS mouse models

**1. Authors**	**2. Age**	**3. Change**	**4. Magnitude of change**	**5. Calculated effect size**	**6. Grouping unit**	**7. Blinding**	**8. Power analysis**	**9. Size parameter**	**10. Sex/no. of animals (WT, *G93A*)**
Pullen and Athanasiou (2009)	P42P70P156	NCIncreaseIncrease	N/A25%56%	N/A0.711.42	By bouton	Not reported	Not reported	Appositional length	M^2^
Herron and Miles (2012)	P8P30P120P140	NCIncreaseIncreaseIncrease	N/A7%14%17%	Inadequate information^1^	Byanimal	Blindingincluded	Not reported	Appositional length (Feret’s diameter)	M (25 mice total for all time points and groups)^3^
Saxena et al. (2013)	P37	Increase	100%	Inadequate information^1^	By bouton	Not reported	Not reported	Volume	M (3, 3)
Milan et al. (2015)	P10P21P40P75P100	DecreaseIncreaseNCNCDecrease	9%7%N/AN/A5%	0.150.17N/AN/A0.43	Bycell	Blindingincluded	Not reported	Area	M (6, 6)M (7, 6)M (8, 6)M (6, 7)M (5, 8)
This article	P10P30P90P120+	NCNCNCNC	N/A	N/AN/AN/AN/A	Bycell	Blindingincluded	Included	Largest cross-sectional area	M (3, 3)M (4, 4)M (3, 3)M (3, 3)

1, Authors; 2, age of *G93A* mouse studied; 3, whether C-bouton size increases, decreases, or there was no change were reported for each age studied; 4, magnitude of change; 5, calculated effect size, if possible; 6, the sampling strategy (grouping unit) used; 7, the reported blinding status, if reported; and 8, the power analysis, if reported. M, Male; N/A, not available; NC, no change.

^1^Inadequate information. SD values or sample size (*n*) data were not included in the published work, making the calculation of effect size impossible.

^2^No information on animal number was provided.

^3^Information on each time point was not provided. Female data not included in the table comparison.

We used immunohistochemistry and confocal microscopy to examine C-boutons in great detail at several stages throughout disease progression. We performed histological analysis to test whether changes in C-bouton size took place, using standard published techniques (Pullen and Athanasiou, 2009; Herron and Miles, 2012; Saxena et al., 2013; Milan et al., 2015). We then performed additional examinations of C-boutons while varying methods of experimental design and data analysis to identify potential sources of research design variability and bias that might impact the results. Last, we examined the possible contribution of research design variability and bias to the conflicting results reported in experimental and preclinical studies of ALS.

Our results show that C-bouton size does not change throughout the disease. Additionally, our results show that variations in the methods of data breakdown and sampling strategy, as well as whether blinding was used during analysis, has significant impact on the outcomes of statistical analysis, such that a statistically significant increase or decrease, or no change, in C-bouton size could all be produced from the same dataset. We concluded that these issues likely contribute to conflicting reports on C-bouton changes in the ALS literature, and that the standardization of experimental design and data analysis will benefit the field.

## Materials and Methods

All animal procedures were performed in accordance with the regulations of the Wright State University Laboratory Animal Care and Use Committee (LACUC).

### Animal genetic background

All mice were either purchased from The Jackson Laboratory or bred from these mice to produce male mice with a B6SJL-TG genetic background (Tg(SOD1*G93A)1Gur). Briefly, B6/SJL hybrid females were bred with male hemizygote mice expressing the human *SOD1* gene with a glycine-to-alanine mutation at amino acid 93 (*SOD1-G93A*). Male offspring of this pairing were used for all experiments and compared with their noncarrier littermates. Because a major goal of our study was to compare our results with a large body of literature, we used males in the present study because ALS studies looking at C-bouton changes have largely used males, and in humans it appears that males are affected more by ALS than females (McCombe and Henderson, 2010). Genotyping using tail clippings was performed by Transnetyx. All mutant hemizygous mice expressed a high copy of the mutated gene (>25 copies). Mutant mice and their noncarrier littermates were killed at four time points [postnatal day 10 (P10), P30, P90, and end-stage], which were predefined at full hindlimb paralysis (∼P120–P140).

### Animal surgical procedures

All animals were anesthetized with EUTHASOL solution (pentobarbital sodium and phenytoin sodium) and transcardially perfused with a vascular rinse (0.01 m phosphate buffer with 0.8% NaCl, 0.025% KCl, and 0.05% NaHCO3, pH 7–8), followed by 4% paraformaldehyde in 0.1 m phosphate buffer, pH 7–8.

### Tissue preparation

The lower lumbar spinal cord was quickly removed and postfixed in 4% paraformaldehyde fixative for ∼2 h or overnight. Tissue was then stored in 15% sucrose at 4°C overnight. Transverse sections of L4–L6 spinal cords were then cut on a cryostat at a thickness of ∼50 μm and collected in 0.01 m PBS, pH 7–8.

### Spinal cord immunohistochemistry

Sections were rinsed with PBS-T (0.01 m PBS containing 0.1% Triton-X, pH 7.3), blocked with normal horse serum (10% in PBS-T), and then incubated free floating in cocktails of primary antibodies overnight at 4°C. All antibodies were diluted with PBS-T. Nissl immunocytochemistry was performed using a 435/455 blue fluorescent Nissl stain (1:100; catalog #N-21479, Neurotrace, Life Technologies) to visualize cell bodies. Labeling of C-boutons was performed with anti-vesicular acetylcholine transporter (VAChT; 1:1000 dilution; goat, Abcam; RRID: AB_956453). All primary antibodies were diluted in PBS-T 0.1%, pH 7.4, and incubated overnight at 4°C. The sensitivity and specificity of the primary antibodies against VAChT have been demonstrated previously (Alvarez et al., 1998, 1999; Deng and Fyffe, 2004; Muennich and Fyffe, 2004; Deardorff et al., 2013). Immunoreactivity was detected with a goat-specific secondary antibody conjugated to Alexa Fluor 647 (Jackson ImmunoResearch) diluted 1:50 in PBS-T 0.1%, pH 7.4, and incubated at room temperature for ∼3 h. Sections were then mounted onto slides and coverslipped in Vectashield mounting medium (Vector Laboratories).

### Confocal microscopy and bouton analysis

Images were obtained on a Fluoview 1000 (Olympus) confocal microscope with a 60× oil-immersion objective in 1 μm steps. Alpha-motoneurons were differentiated on the basis that soma size measurements fell within a previously published range of >300 μm^2^ (Ishihara et al., 2013), that they were located in Rexed lamina IX, and that they received synaptic input from large cholinergic boutons, as evidenced by VAChT immunoreactivity (VAChT-IR). A 2-D analysis of motoneurons was then performed. This process allowed for the relatively efficient analysis of the somatic morphology and neurochemistry of a large sample of motoneurons from each age and genotype group. Bouton areas were measured using Fluoview software. Regions of interest were drawn around the largest cross-sectional area of boutons on every cell.

#### Bouton size measurements

Bouton areas were obtained at four time points (P10, P30, P90, and P120+/end-stage) in *G93A* mutants and their wild-type littermates. One to seven boutons were randomly analyzed per motoneuron, and their properties were averaged by cell for statistical analysis. This method allowed us to measure a large number of en fosse boutons from every cell (i.e., large-sampling strategy), which we maintained throughout our work except when otherwise noted. Two to 16 spinal cord sections per animal were analyzed (*n* = 2–16) from three to five animals per age per genotype (*N* = 3–5). Our data analysis was blinded by coding image genotype information throughout the study except when mentioned otherwise.

#### Bouton number and density measurements

Density measurements were performed according to modified protocols from the study by Alvarez et al. (2011). Briefly, Nissl-stained cells stained for VAChT-IR were randomly sampled from both WT and *G93A* motoneurons. Each cell was imaged with a separation by 1 µm *z*-steps. From these image stacks, a mid-somatic region was identified by the presence of a well defined nucleolus; and from this center image, three optical sections separated by 2 µm in the *z*-axis (to avoid resampling the same terminals) were chosen for quantification. The files containing the sections were then labeled with a letter identifier, and the analyzer was blinded to the genotype. The number of VAChT-IR clusters on the surface of labeled Nissl cell bodies was counted, and the largest cellular perimeter (at the mid-somatic region) for each cell was measured, excluding the origins of primary dendrites. Counts and perimeter measurements were obtained with Fluoview software. Densities were estimated as the number of contacts per 100 µm of linear perimeter. An average density estimate was obtained for each motoneuron sampled.

### Statistical analysis

Statistical analyses were experimental, not descriptive; random sampling was performed. Statistica and GraphPad Prism 6/7 were used for all statistical analyses. Two-way ANOVA, followed by Tukey’s *post hoc* tests, was performed to examine the effects of genotype and age on C-boutons. For ANOVA, *F* values are provided to indicate the significance of the effects. For Mann–Whitney *U* test analysis, *U* values are provided to show the difference between the two rank totals. Significance for all tests was set at *p* ≤ 0.05.

### Analysis of ALS literature

A PubMed search was conducted for all articles containing the term “*G93A*” between May 1, 2015, and May 1, 2016. Any articles that were written in English and available for download through Wright State University libraries was included for analysis. Review articles were excluded, and research articles containing a significant preclinical component were noted. A database was compiled to note the presence or absence of various components of experimental design, including blinding, power analysis, and effect size. The journal name was denoted for all articles used in the analysis.

## Results

To examine C-bouton size in ALS, we used the VAChT-IR to label and measure C-boutons in the ventral horn of spinal cords of *G93A* versus WT mice at various disease stages. We analyzed the following four time points: P10, P30, P90, and end-stage (P120+). At P10, many electrical and morphological motoneuron abnormalities have been observed in the *G93A* model, yet neurodegeneration has not started (Quinlan et al., 2011; Leroy et al., 2014). P30 is early adulthood and early disease stage; and P90 is full adulthood and late disease stage. Specifically, neurodegeneration of fast-type motoneurons starts at P30 followed by the slower types at ∼P90 in this model (Pun et al., 2006; Hegedus et al., 2007, 2008). The end stage of disease in this model, when mice have developed full paralysis of both hindlimbs and fail to right themselves, occurs between P120 and P140, which is designated as P120+. Importantly, these four time points also parallel comparable disease stages of several C-bouton studies in ALS, allowing us to compare our results to the *G93A* line literature (Table 1).

### C-bouton size is not different between WT and G93A motoneurons

**Figure 1.**
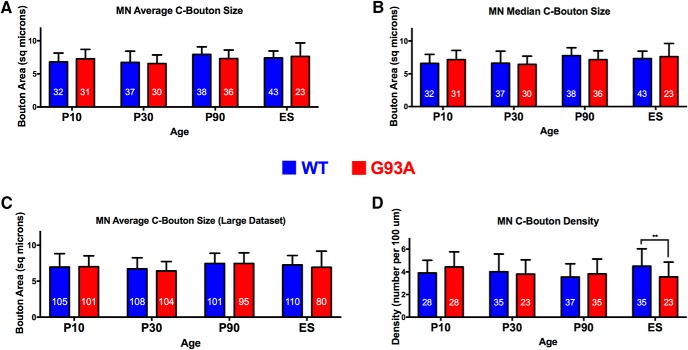
The size of a C-bouton synapse does not change in ALS (all sizes presented in square micrometers). ***A***, Mean C-bouton size values per cell at four time points in WT vs *G93A* motoneurons, with the number of cells sampled per group inside each bar. ***B***, Median C-bouton size values per cell at four time points in WT vs *G93A* motoneurons, with the number of cells sampled per group inside each bar. ***C***, Mean C-bouton size values per cell at four time points in WT vs *G93A* motoneurons, using a larger sample size with >99% statistical power and with the number of cells sampled per group inside each bar. ***D***, Mean C-bouton density values per cell at four time points in WT vs *G93A* motoneurons, with the number of cells sampled per group inside each bar. See Results.

For each time point, we compared data from *G93A* mice to data from age-matched, littermate WT mice. We found no significant differences in the mean cross-sectional area of WT versus *G93A* C-boutons (Fig. 1*A*). We used two-way ANOVA to examine the effects of both genotype and age on bouton size. This analysis revealed a significant effect of age (*F*_(3,262)_ = 7.066; *p* = 0.0001^a^; Table 2) but no genotype or interaction effects. The age effect reflects the normal development of these boutons with age and is in agreement with the published literature (Wetts and Vaughn, 2001). Tukey’s *post hoc* analysis showed a significant increase in mean bouton size between motoneurons at P10 and P30 versus motoneurons at P90 in WT mice (Fig. 1*A*). However, Tukey’s *post hoc* analysis revealed no significant changes in mean bouton size in WT versus *G93A* motoneurons at any time point. To confirm these findings and to minimize the effect of data variability or outliers, we repeated the two-way ANOVA on the median data as opposed to the mean data (Fig. 1*B*), which confirmed the significant effect of age (*F*_(3,262)_ = 7.027; *p* = 0.0001)^b^ and the lack of genotype or interaction effects. Tukey’s *post hoc* analysis revealed no significant changes in C-bouton size between WT and mutant *G93A* motoneurons (Fig. 1*B*). In these statistical analyses, we used a number of cells per group (*n* = 23–43 per group; Fig. 1*A*,*B*), comparable to the number used in most C-bouton ALS studies in the literature (Pullen and Athanasiou, 2009; Herron and Miles, 2012; Saxena et al., 2013), which had statistical power of ∼70%. We reasoned that a larger number of cells might provide sufficient statistical power to reveal a C-bouton size change in WT versus *G93A* cells. Therefore, we increased the sample size of WT and *G93A* cells (*n* ranged between 80 and 110 cells/group), which increased statistical power to 99.4%. Importantly, we saw no differences in C-bouton size between *G93A* and WT at any time point (*p* = 0.21)^c^ (Fig. 1*C*), confirming the analysis conducted in Figure 1*A* at lower statistical power. Statistical power analysis is performed to determine the sample size needed for a statistical test to detect a statistically significant difference when such a difference actually exists; a statistical power of 80% is generally accepted as sufficient.

**Table 2.** Statistical table

**Statistical values from text**	**Data structure**	**Type of test**	**Power**
a. Significant effect of age, mean:(*F*_(3,262)_ = 7.066; *p* = 0.0001)	Normal distribution (8/8 datasets)	Two-way ANOVA	70%
b. Significant effect of age, median:(*F*_(3,262)_ = 7.027; *p* = 0.0001)	Mostly normal distribution (7/8 datasets)	Two-way ANOVA	70%
c. No significant effect of genotype in large-size study, (*F*_(1,796)_ = 1.575; *p* = 0.21)	Mostly normal distribution (5/8 datasets)	Two-way ANOVA	99.4%
d. Significant effect of interaction of age and genotype, mean bouton number:(*F*_(3,243)_ = 6.794; *p* = 0.0002)Uncorrected Fisher’s LSD *post hoc* for P120+ comparison: mean difference, 1.802; *p* < 0.0001; effect size, 1.07	Mostly normal distribution (5/8 datasets)	Two-way ANOVA with Fisher’s LSD *post hoc*	Genotype: 68%
e. Significant effect of interaction of age and genotype, mean bouton density:(*F*_(3,236)_ = 3.346; *p* = 0.0199)Uncorrected Fisher’s LSD *post hoc* for P120+ comparison: mean difference, 0.9378; *p* = 0.0098; effect size, 0.66	Normal distribution (7/8 datasets)	Two-way ANOVA with Fisher’s LSD *post hoc*	Genotype: 68%
f. Per cell analysis shows C-bouton decrease by ∼8%; effect size, 0.51; *p* = 0.0337	Normal distribution (2/2 datasets)	Mann–Whitney *U* test	85%
g. Per bouton analysis shows C-bouton decrease by ∼8%; effect size, 0.33; *p* = 0.0001	Non-normal distribution (2/2 datasets)	Mann–Whitney *U* test	99.9%
h. Large-sampling strategy shows C-bouton decrease by ∼8%; effect size, 0.51; *p* = 0.0337	Normal distribution (2/2 datasets)	Mann–Whitney *U* test	85%
i. Small-sampling strategy shows C-bouton increase by ∼16%; effect size, 0.36; *p* = 0.0039	Non-normal distribution (one-half datasets)	Mann–Whitney *U* test	99.9%
j. Small-sampling strategy, reduced cell count, shows C-bouton increase by ∼28%; effect size, 0.89; *p* = 0.0038	Normal distribution (2/2 datasets)	Mann–Whitney *U* test	70%
k. Unblinded analysis of C-bouton size shows decrease by ∼20%; effect size, 1.22; *p* = 0.0017	Normal distribution (2/2 datasets)	Mann–Whitney *U* test	94.4%

Column 1 supplies the reference letter for those used in the Results section, a description of the effect measured, and the value of the statistical analysis conducted; column 2 states the data structure for that dataset; column 3 states the type of analysis; and column 4 states the statistical power for that dataset analysis.

We have also examined the number and density of C-boutons at different disease stages. The two-way ANOVA showed no effects of genotype or age but showed a significant effect of the interaction between them on bouton number and density. Fisher’s least significant difference (LSD) *post hoc* analysis revealed a significant decrease in C-bouton number (data not shown) and density (Fig. 1*D*) in WT versus *G93A* motoneurons only at end stage, which is in agreement with data from ALS patients (Nagao et al., 1998) and with data from two *G93A* studies (Chang and Martin, 2009; Gallart-Palau et al., 2014), but is opposite to data from other studies reporting either an increase or no change at end stage (Pullen and Athanasiou, 2009; Herron and Miles, 2012).

In sum, our data could not replicate reported differences in the *G93A* line (Pullen and Athanasiou, 2009; Herron and Miles, 2012; Saxena et al., 2013; Milan et al., 2015) by showing no change in C-bouton size, and also showed a decrease in C-bouton number and density only at end stage.

### C-bouton data breakdown, sampling strategy, and blinding influence the statistical analysis outcome

In this section, we examined the potential reasons that could (1) underlie our failure to replicate the published changes on C-bouton size and (2) explain the inconsistency in published data on this topic.

### Grouping unit

**Figure 2.**
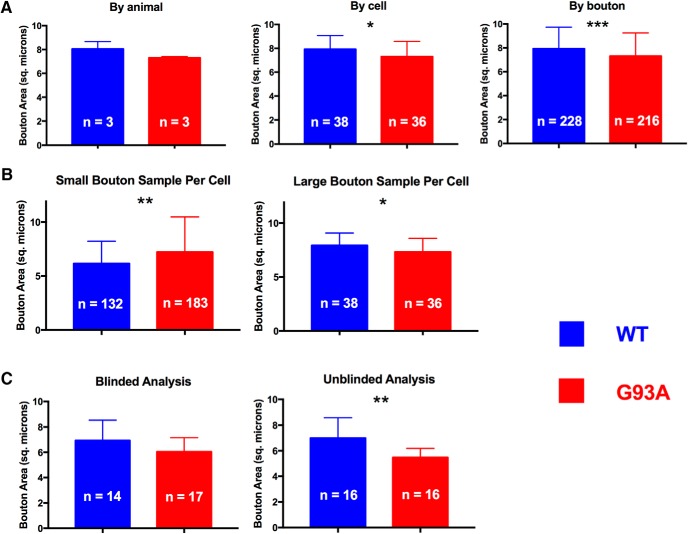
Experimental design considerations make a significant impact on C-bouton differences (all sizes presented in square micrometers). ***A***, Analysis of C-bouton size values by animal (no difference detected), by cell (significant decrease in *G93A* vs WT), and by bouton (significant decrease in *G93A* vs WT) at P90 in WT and *G93A* motoneurons. ***B***, Analysis of C-bouton size values using a small-sampling approach (∼2.5 boutons/cell, a significant increase in *G93A* vs WT motoneurons) vs a large-sampling approach (∼5 boutons per cell, a significant decrease in *G93A* vs WT motoneurons) at P90 in WT and *G93A* motoneurons. ***C***, Blinded (no difference detected) vs unblinded (significant decrease in *G93A* vs WT motoneurons) analysis of C-bouton size values at P30 in WT and *G93A* motoneurons (*, **, and *** indicate significant results). See Results.

Data on C-bouton size have been reported and analyzed in the literature by different units (average area per animal, average area per cell, or average area per bouton; Table 1). Thus, we examined the possibility that different methods of data breakdown could contribute to the discrepancy in published results. We further analyzed the P90 time point (an advanced stage of disease, where we might expect more detectable disease changes) and compared C-bouton cross-sectional area in WT versus *G93A* cells. Data were broken down and averaged in three different ways, as follows: (1) by animal; (2) by cell; or (3) by bouton (Fig. 2*A*). In these analyses, we used the Mann–Whitney *U* test because: (1) only a single time point is being considered; and (2) the *U* test does not require a normal distribution of data, making it more general than a Student’s *t* test. Generally, statistical analysis generated different results among the three breakdown strategies, despite comparable average areas being examined in each breakdown (Fig. 2*A*, all blue bars have similar magnitudes, as do all red bars). Although the Tukey’s *post hoc* analysis of the two-way ANOVA discussed in first section of Results indicated no statistical difference in WT versus *G93A* cells at the P90 time point (Fig. 1*A*), in the per cell analysis, the Mann–Whitney *U* test showed a significant decrease in *G93A* bouton size (by ∼8%; effect size, 0.51; *p* = 0.0337^d^; statistical power, ∼85%; Fig. 2*A*, middle bars). This difference was not detected by the stringent *post hoc* Tukey’s test in Figure 1*A*, probably due to its small magnitude. The “per bouton” analysis showed a similar significant decrease in *G93A* bouton size (by ∼8%; effect size, 0.33; *p* = 0.0001^e^; statistical power, 99.9%; Fig. 2*A*, right bars). Conversely, the “per animal” analysis indicated no statistical difference in C-bouton size in WT versus *G93A* motoneurons (statistical power, 13%, Fig. 2*A*, right bars). This discrepancy in statistical analysis outcome is due to the large difference in sample size (i.e., the *n*) for each group based on the method of data breakdown (the *n* for each group is shown in the bars of Fig. 2). These results demonstrate that the method of data breakdown could strongly influence the outcome of statistical analysis and, thus, the reported results.

### Sampling strategy

We also examined whether the strategy of bouton sampling from cells influences statistical analysis, again using the Mann–Whitney *U* test for the following analyses. We examined two strategies of sampling boutons per cell: (1) large sampling of boutons per cell (three to seven boutons measured per cell; average, 5 boutons/cell; Fig. 2*B*, right bars); and (2) small sampling of boutons per cell (one to three boutons measured per cell; average, 2.5 boutons/cell; Fig. 2*B*, left bars). Because the small-sampling strategy generated a much smaller number of boutons than the large-sampling strategy, we collected more cells using the small-sampling strategy to ensure rigorous statistical analysis (cell *n* = 132 for WT cells, and *n* = 183 for *G93A* cells; Fig. 2*B*). When WT and *G93A* data were compared, the large-sampling strategy showed a statistical decrease (by ∼8%; effect size, 0.51; *p* = 0.0337^f^; statistical power, ∼85%; Fig. 2*B*, right bars; cell *n* = 38 for WT cells and *n* = 36 for *G93A* cells) in *G93A* C-bouton size, whereas the small-sampling strategy showed a statistical increase in *G93A* C-bouton size (by ∼16%; effect size, 0.36; *p* = 0.0039^g^; statistical power, 99.9%; Fig. 2*B*, left bars). To confirm that the imbalance in the number of cells between the two strategies is not responsible for this discrepancy in analysis outcome, we reanalyzed a smaller number of small-sampling cells (cell *n* = 27 for WT cells and *n* = 26 for *G93A* cells, which is comparable to the number of cells analyzed in the large-sampling strategy), selected randomly from the same cells originally analyzed. This reanalysis resulted in a significant increase in *G93A* C-bouton size that was comparable to the initial small-sampling analysis (by ∼28%; effect size, 0.89; *p* = 0.0038^h^; statistical power, ∼70%; data not shown). This indicates that it is the sampling strategy, not the number of cells, that reversed the outcome of the small-sampling statistical analysis. These results demonstrate that the way C-boutons are sampled from cells can significantly influence the outcome of the statistical analysis and, thus, the conclusions drawn.

### Blinding

Blinding is the process of having the experimenter analyze data without prior knowledge of whether the data belong to the control group or the experimental group. Although blinding is recommended in data analysis to minimize potential bias, we found that this practice is not commonly reported in ALS literature. For instance, Table 1 shows that on the topic of C-bouton size change, only two among five recent studies explicitly mentioned that they blinded their analysis. It is unknown whether the other three studies performed a blinded analysis. It is interesting to observe that the magnitude of the reported changes and the effect size were generally much smaller when blinding was reported (Table 1). Therefore, we examined whether blinding the analyzer would have an effect on the outcome of analysis. We asked one experimenter to analyze a random subset of cells of the P30 time point data while blinded to animal genotype. No statistical difference was observed in WT versus *G93A* C-bouton size using the Mann–Whitney *U* test (Fig. 2*C*, left). However, when the same experimenter was asked to analyze the same slices, among other slices, while knowing which slices belonged to which genotype, we observed a statistically significant reduction in C-bouton size (by ∼20%; effect size, 1.22; *p* = 0.0017)^i^ in *G93A* data relative to WT data (Fig. 2*C*, right). Importantly, the experimenter did not know that the impact of blinding was being studied when these analyses were conducted. Notably, the magnitude of the detected decrease in C-bouton size and its effect size was largest under unblinded conditions versus our other analyses that showed a decrease in C-bouton size [20% decrease of effect size of 1.22 under unblinded conditions (Fig. 2*C*, right) vs 8% decrease of effect size of 0.51 with a large-sampling strategy (Fig. 2*B*, right) vs 8% decrease of effect size of 0.33 with per bouton analysis (Fig. 2*A*, right)]. These results demonstrate that blinding status can have significant impact on the outcome of data analysis. Taken collectively, these results demonstrate that methods of data breakdown, sampling strategies, and blinding can significantly influence the outcome of statistical analysis, which could contribute to the discrepancy in published results on C-bouton changes in ALS.

### Reporting of blinding status is not the norm in ALS experimental and preclinical studies

**Figure 3:**
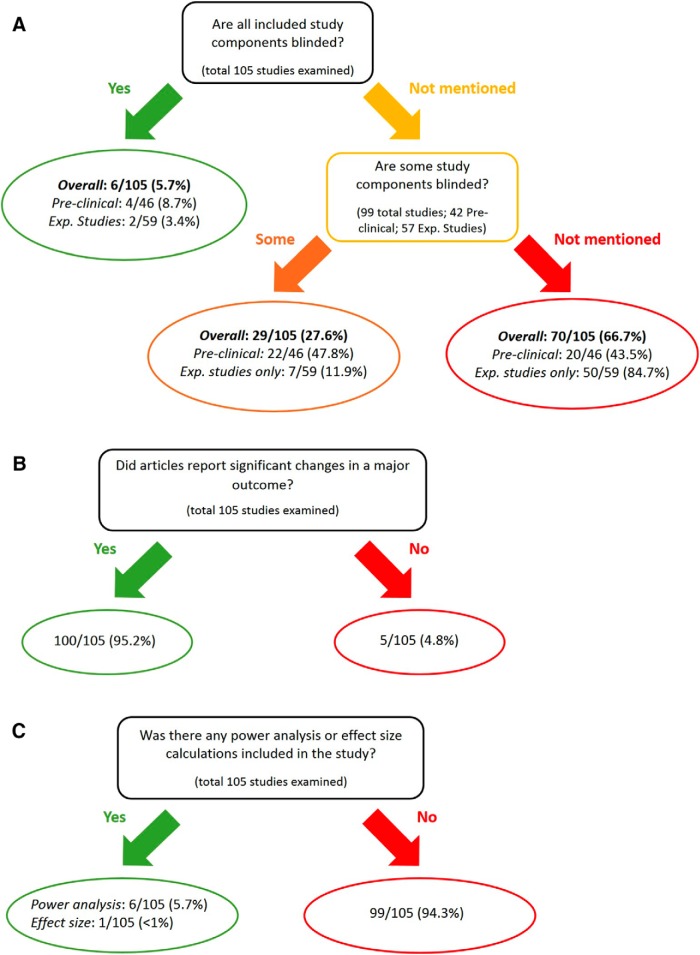
, The majority of ALS studies do not report blinding and/or power analysis. ***A***, The number of ALS studies on the *G93A* model conducted between May 2015 and May 2016 that reported blinding of their analysis vs those that did not report blinding [6 studies (5.7%) reported all elements blind, 29 studies [27.6%] reported some elements blind, 70 studies (66.7%) did not report blinding]. The analysis is further broken down by studies containing a preclinical component vs purely experimental studies. ***B***, The number of ALS studies that reported statistically significant results vs those that reported no changes [100 studies (95.2%) reported significant results, 5 studies (4.8%) did not]. ***C***, The number of ALS studies that reported power analysis and/or effect size calculations vs those that did not report these data [6 studies (5.7%) reported power analysis, 1 of these (<1%) reported effect size as well, and 99 studies (94.3%) reported neither). See Results.

Given our results on the strong impact of study design variability and the potential bias on reported data, we wanted to examine whether these issues are prevalent in the experimental and preclinical studies of ALS. To achieve that, we analyzed published experimental and preclinical articles between May 2015 and May 2016 that studied ALS using the *G93A* model, which resulted in 105 articles (Fig. 3*A*). The analysis showed that 6 of 105 experimental and preclinical articles reported blinding in all of their measurements (5.7%). Of those, 4 of 46 articles included a preclinical component and reported blinding of all of their measurements (8.7%), whereas 2 of 59 articles did not include a preclinical component or were solely experimental and reported blinding of all of their measurements (3.4%; Fig. 3*A*, green block). Importantly, 29 articles reported blinding in some of their measurements (27.6%; Fig. 3*A*, orange block). Of those, 22 articles included a preclinical component (47.8%), whereas 7 articles did not include a preclinical component or were solely experimental (11.9%). Strikingly, a large percentage of the articles (70 of 105 articles) had no mention of blinding in any of their measurements (66.7%; Fig. 3*A*, red block). Of those, 20 articles (43.5%) had a preclinical component, whereas 50 articles did not include a preclinical component or were solely experimental (84.7%). This analysis surprisingly suggests that blinding might not be a common practice in the ALS literature.

### Reports of significant results and datasets without reported power analysis are prevalent in ALS experimental and preclinical research

Our analysis also revealed that 100 of 105 articles reported a significant result in a major outcome measure (95.2%; Fig. 3*B*), whereas 5 articles (4.8%) reported no significant result from a major outcome measure. In addition, 99 of the 105 articles (94.3%) reported no statistical power analysis in support of the sample sizes used to determine significant results (Fig. 3*C*). Six of the 105 articles (5.7%) reported a power analysis; and 1 article reported effect size (0.95%) as well.

## Discussion

The present study examines potential factors that might contribute to the conflicting data reported on the disputed topic of C-bouton size changes in ALS and additionally examines this topic in our own large-sample, statistically robust study. We examined (1) C-bouton size, number, and density in WT versus *G93A* mice at several time points over the full span of disease progression in *G93A* mice, which serves as a model for ALS in human patients; (2) collected large WT and *G93A* data samples and conducted power analysis and effect size analysis; (3) examined different methods of sampling and data analysis; and (4) examined the impact of blinding. Our data showed that different statistical outcomes (enlargement, no change, or diminution) in C-bouton size could be produced from the same dataset of WT and *G93A* groups, depending on which experimental approaches and data analysis practices were used. We also examined effect size and power analysis, where possible, in the studies we assessed.

C-boutons are cholinergic synapses that have been suggested to increase spinal motoneuron excitability through the regulation of ion channels responsible for changes in firing rate (Wilson et al., 2004; Brownstone, 2006; Miles et al., 2007). Thus, any putative changes in these inputs (e.g., size, density per cell, activity) would appear to have tremendous implications for the firing behavior of motoneurons during disease conditions in ALS. It seems appropriate, then, that a great deal of importance was placed on the repeated detection of morphological abnormalities in these boutons on motoneurons from *G93A* mutant mice in past studies (Table 1). In our opinion, the failure of our own study to detect any changes in C-bouton size does not in any way decrease their significance to motoneuron excitability or their possible role in pathological states.

### C-bouton size does not differ between WT and G93A motoneurons

In contrast to a number of published reports, our analysis of C-bouton size did not show differences between WT and *G93A* motoneurons. This was surprising, as bouton enlargement has been reported in the literature (Pullen and Athanasiou, 2009; Herron and Miles, 2012; Saxena et al., 2013; Milan et al., 2015) and has been interpreted as an example of an ALS disease change that influences motoneuron excitability. It is important to mention that our investigation, as opposed to most studies on this topic (Table 1), was based on large samples of WT and *G93A* motoneurons supported by statistical power analysis and was conducted with blinding to avoid potential bias. We also followed a large-sampling strategy in collecting bouton data per cell.

### C-bouton number and density do not differ between WT and G93A motoneurons except at end stage

Similar to the size of C-boutons, their number and density is another disputed topic in the ALS field in which inconsistent results of increase (Pullen and Athanasiou, 2009; Herron and Miles, 2012; Vinsant et al., 2013; Milan et al., 2015), decrease (Chang and Martin, 2009; Casas et al., 2013; Gallart-Palau et al., 2014; Milan et al., 2015; Vaughan et al., 2015), and no change (Chang and Martin, 2009; Pullen and Athanasiou, 2009; Herron and Miles, 2012; Casas et al., 2013; Gallart-Palau et al., 2014; Milan et al., 2015; Vaughan et al., 2015) have been reported at different disease stages, sometimes all in the same study. Importantly, postmortem data available from ALS patients show a decrease in C-bouton number at end stage (Nagao et al., 1998). Our data agree with the human results and constitute one of three datasets that suggest there is a depletion of C-boutons only at the end stage (Chang and Martin, 2009; Gallart-Palau et al., 2014). Additionally, we do not see any change (increase or decrease) at earlier time points, contrary to a number of nonhuman studies. While there may be some inherent shortcomings with the animal models, inconsistent results on the same topic in the same animal model raise serious questions about the rigor of experimental design and methods of data analysis that probably contribute to unsuccessful translation (Gordon and Meininger, 2011). It is possible that widespread adoption of consistent experimental design and data analysis methods will help to clarify the comparative value of various animal models to human pathology.

#### Experimental design considerations

Our results indicate that several experimental design factors could influence statistical analysis sufficiently to produce different outcomes from the same dataset.

#### Grouping unit

C-bouton size data have been reported in the ALS literature using different grouping units (by animal, by cell, or by bouton; Table 1). Our analysis indicates that changing how the data are grouped has a large impact on the significance level and outcome of the statistical analysis, even with no change in the raw averages. This effect comes from the fact that, for a given dataset, the grouping unit determines the sample *n* size (Fig. 2*A*, the sample size per bouton > sample size per cell > sample size per animal), which goes into the calculation of the significance level and, therefore, influences whether a difference could be detected between the control and experimental groups. Accordingly, this issue raises the question of the proper grouping unit to be used when analyzing and comparing data. We suggest using the cell as the grouping unit and as the basis of comparison between the control and experimental groups. Cells are known to be of different types (e.g., slow vs fast motoneurons) and could be affected differently in diseases. Thus, it is plausible to expect boutons of different cell types to be affected differently, and, therefore, it becomes important not to pool boutons of all cell types. On the other hand, grouping data by animal averages lacks the adequate statistical power needed for rigorous statistical analysis.

#### Sampling strategy

Our analysis indicates that the method for sampling boutons per cell has a significant effect on the outcome of statistical analysis, such that a statistical increase between the control and experimental groups could be reversed to a decrease (Fig. 2*B*, an example). This factor is challenging, because most published studies do not include information on how they sampled their measurements. This issue, therefore, raises another question on the proper sampling strategy to be used when collecting bouton data from cells. We recommend collecting many boutons per cell (i.e., a large-sampling strategy) because this approach considers any potential intracellular variability among the boutons and makes the data less sensitive to errors and outliers. One potential explanation for the conflicting data in ALS literature on C-bouton size could be due to differences among studies in the way boutons have been sampled from motoneurons. If a small-sampling strategy was followed (i.e., collecting few boutons per cell), this could underlie and explain the increase in C-bouton size that has been observed in several, but not all, C-bouton studies in ALS (Table 1). Similarly, unbiased stereology practices (e.g., random sampling of regions of interest and precise rules for marking and quantifying samples) could potentially influence analyses and should be considered and strictly applied in order to ensure unbiased quantification.

#### Blinding

A key issue that impacts potential bias in measurements is blinded versus unblinded data analysis. A comparison of both conditions led us to the conclusion that a lack of blinding can result in false-positive data. While raw measurements were not dramatically different between blinded and unblinded datasets, we saw statistically significant differences between groups when the assessor knew which group was experimental and which group was the control; while there were no statistically significant differences between groups when the same assessor performed the analysis in blinded conditions (Fig. 2*C*). This suggests that bias might affect results if the data analysis is performed without blinding. Although a number of C-bouton studies have blinded their analysis and have reported that explicitly, we found that many studies have not included any information on whether blinding was followed in analyzing their data (Table 1). Interestingly, the magnitude of reported changes and the effect size were generally much smaller in studies in which blinding was reported versus those in which blinding was not reported (Table 1). Importantly, our analysis showed that 94.3% of the examined ALS studies, preclinical and basic, do not report blinding or other procedures to limit bias. While there are no data on under-reporting that provide statistics on what percentage of studies do not report, but do perform, blinding, it is puzzling why authors would omit this important detail from their published work, if implemented. The data from our analysis do show a correlation between studies that report blinding and studies that report a small magnitude of significant results and a small effect size (Table 1).

#### Effect size and power analysis

The effect size and power analysis are two important statistical parameters that quantify the magnitude of a given change and whether the sample used is adequate to detect this change with confidence, and we recommend that these be consistently calculated and reported. Despite the importance of these parameters, we found that all C-bouton studies listed in Table 1 did not include these data. We therefore attempted to calculate the effect size of these studies from their published data; then we compared this information to our effect size data to assess the magnitudes of the reported differences. Importantly, we could not calculate the effect size for three of five studies due to a lack of information on either the SD or the sample size (Table 1). Of the two studies for which we were able to calculate the effect size, one had small effect size values, indicating a small effect of the reported changes (Milan et al., 2015; Table 1), and the second study had relatively high effect size values, although the sample size was a less than one-quarter of our dataset (sample sizes of 17–22 in Pullen and Athanasiou, 2009 vs our sample size of 80–110 in the large dataset seen in Fig. 1*C*). Strikingly, none of the studies included any power analysis to assess whether the sample size was adequate to detect differences with confidence. The lack of power analysis in all studies, combined with either a small effect size or an unsupported large effect size makes it difficult to assess the scientific significance of the reported findings on C-bouton size in ALS. This conclusion is in agreement with similar observations by Scott et al. (2008) on ALS preclinical studies. Our results, supported by a power analysis >99%, indicate no changes in C-bouton size in *G93A* motoneurons.

#### Other considerations

There are several other factors among ALS studies that could still impact the reproducibility of results: (1) background strains: although the data we collected in this study and the literature against which we compared our data were all obtained from *G93A* mice of the high-expresser line (with a copy number >25) to normalize the *G93A* expression level, studies in the literature have used different background strains of mice, which have different disease/survival timelines and might have different disease mechanisms (Table 1); (2) mutations: it is possible that transgenic animal models of ALS with different gene mutations (e.g., FUS, TDP-43, G85R, G37R) or a different copy number (e.g., G93A with a low copy number <8) could have disease mechanisms that differ among models or differ from human pathology, leading to inconsistencies in results; (3) size parameter: the C-bouton size has been assessed in literature using different measures (e.g., surface area, largest cross-sectional area, volume, appositional length; Table 1), and notably, in our study, these differing measures produced comparable results (i.e., similar differences and percentage changes between WT and *G93A* mice across methods) in our datasets (data not shown); and (4) sex: ALS studies looking at C-bouton changes have largely used males for their studies, and it appears that males are affected more by ALS than females (McCombe and Henderson, 2010). Because a major goal of our study was to compare our results to the larger body of literature, we preferred to use males in the present study in order to allow closer comparison. It is noteworthy that Herron and Miles (2012) found no difference in C-bouton size in female mice. These factors are important to be considered in order to have successful replication of results.

It is also noteworthy that the experimental design issues discussed here are not unique to ALS research. Similar issues have been observed in the design of studies in cancer, stroke, Parkinson’s disease, and multiple sclerosis (Sena et al., 2007; Hess, 2011). Additionally, a positive correlation was found between studies that do not report the use of practices such as blinding and power analysis with data that is not reproducible (Scott et al., 2008; Landis et al., 2012). Accordingly, guidelines on best practices for conducting ALS research (including recommendations on mouse model and strain use, colony management, sample sizes, blinding, and statistical methods) have been developed (Leitner et al., 2009; Ludolph et al., 2010). Also, funding agencies, such as the National Institutes of Health, now require explicit description of the use of blinding in the proposed research design as well as reporting of the statistical power of the proposed sample sizes and statistical analyses.

### Conclusion

The conflicting results on C-bouton size in the ALS literature makes it difficult to assess the role of this synaptic input in the disease. Our analysis showed that factors related to experimental design, such as the grouping unit, sampling strategy, and blinding, could contribute to and explain the failure in replicating results as well as the discrepancy in published data on this topic. Furthermore, the lack of power analysis and effect size data makes it difficult to assess the scientific significance of the reported findings on this topic. Our analyses, backed by blinding practices, large samples, and power analysis, do show that the size of C-boutons does not change in *G93A* motoneurons throughout the disease. The number and density of C-boutons were found to be reduced only at end stage, which is in agreement with data from ALS patients. We expect that widespread adoption of consistent practices, such as those proposed here, will help to clarify many such disputed topics within both the field of ALS research and in other fields of neuroscience, leading to improved clinical translation of results.

## Synthesis

Reviewing Editor: Robert Kalb, Children's Hospital of Philadelphia Decisions are customarily a result of the Reviewing Editor and the peer reviewers coming together and discussing their recommendations until a consensus is reached. When revisions are invited, a fact-based synthesis statement explaining their decision and outlining what is needed to prepare a revision will be listed below. The following reviewer(s) agreed to reveal their identity: Angela Jablonski, Lyle Ostrow

Both reviewers find the work a useful addition but each has a set of issues that need to be addressed.

Reviewer #1:

Overall, this is a well-written paper discussing a critical need for more standardization in data analysis and data reporting. The paper fits the journal's scope in terms of reporting non-reproducible data and negative results. I do, however, feel that several things are missing from this paper:

-quantification of c-bouton number in wt and G93A animals at various ages

-normalization of G93A expression levels and discussion of how that might impact changes in the reproducibility of this model

-investigation of another ALS model OR some discussion that these inconsistencies may support the notion that G93A mice are not the best workable model for clinical ALS research

Minor comments:

-under figure legends, remove or change "Graph explaining" or "Diagram explaining" to something more explicit as it is clear it is a graph/table/diagram

-include some proposal for how we might standardize statistical and reporting methods in future studies

-discussion that this problem is not unique to this specific area of neuroscience, or to disease research

Reviewer #2

I think this is an important paper, and worthy of publication, but not for the reason that the authors focus on. The conclusion regarding C-Bouton sizes is secondary. The considerably more important finding is that the same data set, when analyzed blinded vs. unblinded, and with different techniques, can lead to different results. This is a recurring theme and a substantial problem in ALS research - especially with small "N" mouse experiments, and likely is directly responsible for innumerable failed translational efforts. Therefore, I would recommend reworking the focus of the article, including changing the title to reflect this. Additionally:

1. I would include a discussion (and references to) the published guidelines on using the ALS mice, such as

1) S Scott et al., Amyotrophic Lateral Sclerosis. 2008; 9:4-15.

2) AC Ludolph et al, Amyotrophic Lateral Sclerosis. 2010; 11:38-45.

3) M Leinter, S Menzies, C Lutz, Woirking with ALS Mice, published by Jackson Labs and Prize4Life.

2. The researchers only used male mice. While this makes the analysis easier, is it possible that C-bouton size changes are significant in female mice?

3. To this end, on Table 1, they should state the sex of the mice used in the other publications that they cite, as well as the number of animals.

4. A minor point, but they suggest that they chose the mouse ages for analysis to reflect the prior literature studies on c-bouton sizes, but this really isn't clear from the studies they reference.

The above issues need to be addressed in a re-submission

## References

[B1] Alvarez FJ, Pearson JC, Harrington D, Dewey D, Torbeck L, Fyffe RE (1998) Distribution of 5-hydroxytryptamine-immunoreactive boutons on alpha-motoneurons in the lumbar spinal cord of adult cats. J Comp Neurol 393:69–83. 10.1002/(SICI)1096-9861(19980330)393:1<69::AID-CNE7>3.0.CO;2-O 9520102

[B2] Alvarez FJ, Dewey DE, McMillin P, Fyffe RE (1999) Distribution of cholinergic contacts on Renshaw cells in the rat spinal cord: a light microscopic study. J Physiol 515:787–797. 10.1111/j.1469-7793.1999.787ab.x 10066905 PMC2269191

[B3] Alvarez FJ, Titus-Mitchell HE, Bullinger KL, Kraszpulski M, Nardelli P, Cope TC (2011) Permanent central synaptic disconnection of proprioceptors after nerve injury and regeneration. I. Loss of VGLUT1/IA synapses on motoneurons. J Neurophysiol 106:2450–2470. 10.1152/jn.01095.2010 21832035 PMC3214094

[B4] Bensimon G, Lacomblez L, Meininger V (1994) A controlled trial of riluzole in amyotrophic lateral sclerosis. ALS/Riluzole Study Group. N Engl J Med 330:585–591. 10.1056/NEJM199403033300901 8302340

[B5] Brownstone RM (2006) Beginning at the end: repetitive firing properties in the final common pathway. Prog Neurobiol 78:156–172. 10.1016/j.pneurobio.2006.04.002 16725251 PMC5061565

[B6] Casas C, Herrando-Grabulosa M, Manzano R, Mancuso R, Osta R, Navarro X (2013) Early presymptomatic cholinergic dysfunction in a murine model of amyotrophic lateral sclerosis. Brain Behav 3:145–158. 10.1002/brb3.104 23531559 PMC3607155

[B7] Chang Q, Martin LJ (2009) Glycinergic innervation of motoneurons is deficient in amyotrophic lateral sclerosis mice: a quantitative confocal analysis. Am J Pathol 174:574–585. 10.2353/ajpath.2009.080557 19116365 PMC2630565

[B8] Charcot J (1874) De la sclerose laterale amyotrophique. Prog Med 2:325–455.

[B9] Deardorff AS, Romer SH, Deng Z, Bullinger KL, Nardelli P, Cope TC, Fyffe REW (2013) Expression of postsynaptic Ca2+-activated K+ (SK) channels at C-bouton synapses in mammalian lumbar α-motoneurons. J Physiol (Lond) 591:875–897. 10.1113/jphysiol.2012.240879 23129791 PMC3591704

[B10] Deng Z, Fyffe RE (2004) Expression of P2X7 receptor immunoreactivity in distinct subsets of synaptic terminals in the ventral horn of rat lumbar spinal cord. Brain Res 1020:53–61. 10.1016/j.brainres.2004.06.014 15312787

[B11] Fischer LR, Culver DG, Tennant P, Davis AA, Wang M, Castellano-Sanchez A, Khan J, Polak MA, Glass JD (2004) Amyotrophic lateral sclerosis is a distal axonopathy: evidence in mice and man. Exp Neurol 185:232–240. 10.1016/j.expneurol.2003.10.004 14736504

[B12] Gallart-Palau X, Tarabal O, Casanovas A, Sábado J, Correa FJ, Hereu M, Piedrafita L, Calderó J, Esquerda JE (2014) Neuregulin-1 is concentrated in the postsynaptic subsurface cistern of C-bouton inputs to α-motoneurons and altered during motoneuron diseases. FASEB J 28:3618–3632. 10.1096/fj.13-248583 24803543

[B13] Gordon PH, Meininger V (2011) How can we improve clinical trials in amyotrophic lateral sclerosis? Nat Rev Neurol 7:650–654. 10.1038/nrneurol.2011.147 21947135

[B14] Gurney ME, Pu H, Chiu AY, Dal Canto MC, Polchow CY, Alexander DD, Caliendo J, Hentati A, Kwon YW, Deng HX, et al. (1994) Motor neuron degeneration in mice that express a human Cu,Zn superoxide dismutase mutation. Science 264:1772–1775. 8209258 10.1126/science.8209258

[B15] Hegedus J, Putman CT, Gordon T (2007) Time course of preferential motor unit loss in the SOD1 G93A mouse model of amyotrophic lateral sclerosis. Neurobiol Dis 28:154–164. 10.1016/j.nbd.2007.07.003 17766128

[B16] Hegedus J, Putman CT, Tyreman N, Gordon T (2008) Preferential motor unit loss in the SOD1G93A transgenic mouse model of amyotrophic lateral sclerosis. J Physiol 586:3337–3351. jphysiol.2007.149286 18467368 10.1113/jphysiol.2007.149286 PMC2538809

[B17] Herron LR, Miles GB (2012) Gender-specific perturbations in modulatory inputs to motoneurons in a mouse model of amyotrophic lateral sclerosis. Neuroscience 226:313–323. 10.1016/j.neuroscience.2012.09.031 23000617

[B18] Hess KR (2011) Statistical design considerations in animal studies published recently in cancer research. Cancer Res 71:625. 10.1158/0008-5472.CAN-10-3296 21239476

[B19] Ishihara A, Nagatomo F, Fujino H, Kondo H, Ohira Y (2013) Decreased succinate dehydrogenase activity of gamma and alpha motoneurons in mouse spinal cords following 13 weeks of exposure to microgravity. Neurochem Res 38:2160–2167. 10.1007/s11064-013-1124-y 23943522

[B20] Landis SC, Amara SG, Asadullah K, Austin CP, Blumenstein R, Bradley EW, Crystal RG, Darnell RB, Ferrante RJ, Fillit H, Finkelstein R, Fisher M, Gendelman HE, Golub RM, Goudreau JL, Gross RA, Gubitz AK, Hesterlee SE, Howells DW, Huguenard J, et al. (2012) A call for transparent reporting to optimize the predictive value of preclinical research. Nature 490:187–191. 10.1038/nature11556 23060188 PMC3511845

[B21] Leitner M, Menzies S, Lutz C (2009) Working with ALS mice In: Guidelines for preclinical testing & colony management. Cambridge, MA: Prize4Life/The Jackson Laboratory, Bar Harbor, ME.

[B22] Leroy F, Lamotte d'Incamps B, Imhoff-Manuel RD, Zytnicki D (2014) Early intrinsic hyperexcitability does not contribute to motoneuron degeneration in amyotrophic lateral sclerosis. Elife 3:e04046 10.7554/eLife.04046 PMC4227046 25313866

[B23] Ludolph AC, Bendotti C, Blaugrund E, Chio A, Greensmith L, Loeffler JP, Mead R, Niessen HG, Petri S, Pradat PF, Robberecht W, Ruegg M, Schwalenstöcker B, Stiller D, van den Berg L, Vieira F, von Horsten S (2010) Guidelines for preclinical animal research in ALS/MND: a consensus meeting. Amyotroph Lateral Scler 11:38–45. 10.3109/17482960903545334 20184514

[B24] McCombe PA, Henderson RD (2010) Effects of gender in amyotrophic lateral sclerosis. Gend Med 7:557–570. 10.1016/j.genm.2010.11.010 21195356

[B25] Milan L, Courtand G, Cardoit L, Masmejean F, Barrière G, Cazalets JR, Garret M, Bertrand SS (2015) Age-related changes in pre- and postsynaptic partners of the cholinergic C-boutons in wild-type and SOD1G93A lumbar motoneurons. PLoS One 10:e0135525. 10.1371/journal.pone.0135525 26305672 PMC4549056

[B26] Miles GB, Hartley R, Todd AJ, Brownstone RM (2007) Spinal cholinergic interneurons regulate the excitability of motoneurons during locomotion. Proc Natl Acad Sci U S A 104:2448–2453. 10.1073/pnas.0611134104 17287343 PMC1794344

[B27] Muennich EA, Fyffe RE (2004) Focal aggregation of voltage-gated, Kv2.1 subunit-containing, potassium channels at synaptic sites in rat spinal motoneurones. J Physiol 554:673–685. 10.1113/jphysiol.2003.056192 14608003 PMC1664801

[B28] Nagao M, Misawa H, Kato S, Hirai S (1998) Loss of cholinergic synapses on the spinal motor neurons of amyotrophic lateral sclerosis. J Neuropathol Exp Neurol 57:329–333. 9600225 10.1097/00005072-199804000-00004

[B29] Pullen AH, Athanasiou D (2009) Increase in presynaptic territory of C-terminals on lumbar motoneurons of G93A SOD1 mice during disease progression. Eur J Neurosci 29:551–561. 10.1111/j.1460-9568.2008.06602.x 19187267

[B30] Pun S, Santos AF, Saxena S, Xu L, Caroni P (2006) Selective vulnerability and pruning of phasic motoneuron axons in motoneuron disease alleviated by CNTF. Nat Neurosci 9:408–419. 10.1038/nn1653 16474388

[B31] Quinlan KA, Schuster JE, Fu R, Siddique T, Heckman CJ (2011) Altered postnatal maturation of electrical properties in spinal motoneurons in a mouse model of amyotrophic lateral sclerosis. J Physiol 589:2245–2260. 10.1113/jphysiol.2010.200659 21486770 PMC3098701

[B32] Rowland LP, Shneider NA (2001) Amyotrophic lateral sclerosis. N Engl J Med 344:1688–1700. 10.1056/NEJM200105313442207 11386269

[B33] Saxena S, Roselli F, Singh K, Leptien K, Julien JP, Gros-Louis F, Caroni P (2013) Neuroprotection through excitability and mTOR required in ALS motoneurons to delay disease and extend survival. Neuron 80:80–96. 10.1016/j.neuron.2013.07.027 24094105

[B34] Scott S, Kranz JE, Cole J, Lincecum JM, Thompson K, Kelly N, Bostrom A, Theodoss J, Al-Nakhala BM, Vieira FG, Ramasubbu J, Heywood JA (2008) Design, power, and interpretation of studies in the standard murine model of ALS. Amyotroph Lateral Scler 9:4–15. 10.1080/17482960701856300 18273714

[B35] Sena E, van der Worp HB, Howells D, Macleod M (2007) How can we improve the pre-clinical development of drugs for stroke? Trends Neurosci 30:433–439. 10.1016/j.tins.2007.06.009 17765332

[B36] Turner BJ, Talbot K (2008) Transgenics, toxicity and therapeutics in rodent models of mutant SOD1-mediated familial ALS. Prog Neurobiol 85:94–134. 10.1016/j.pneurobio.2008.01.001 18282652

[B37] Vaughan SK, Kemp Z, Hatzipetros T, Vieira F, Valdez G (2015) Degeneration of proprioceptive sensory nerve endings in mice harboring amyotrophic lateral sclerosis-causing mutations. J Comp Neurol 523:2477–2494. 10.1002/cne.23848 26136049 PMC5759336

[B38] Vinsant S, Mansfield C, Jimenez-Moreno R, Del Gaizo Moore V, Yoshikawa M, Hampton TG, Prevette D, Caress J, Oppenheim RW, Milligan C (2013) Characterization of early pathogenesis in the SOD1(G93A) mouse model of ALS: part II, results and discussion. Brain Behav 3:431–457. 10.1002/brb3.142 24381813 PMC3869683

[B39] Wetts R, Vaughn JE (2001) Development of cholinergic terminals around rat spinal motor neurons and their potential relationship to developmental cell death. J Comp Neurol 435:171–183. 10.1002/cne.1200 11391639

[B40] Wilson JM, Rempel J, Brownstone RM (2004) Postnatal development of cholinergic synapses on mouse spinal motoneurons. J Comp Neurol 474:13–23. 10.1002/cne.20089 15156576

